# Feeling Touched: Empathy Is Associated With Performance in a Tactile Acuity Task

**DOI:** 10.3389/fnhum.2021.593425

**Published:** 2021-02-09

**Authors:** Michael Schaefer, Marcel Joch, Nikolas Rother

**Affiliations:** Department Naturwissenschaften, Medical School Berlin, Berlin, Germany

**Keywords:** empathy, tactile perception, two-point discrimination threshold, primary somatosensory cortex, touch (haptic/cutaneous/tactile/kinesthesia)

## Abstract

The concept of empathy describes our capacity to understand the emotions and intentions of others and to relate to our conspecifics. Numerous studies investigated empathy as a state as well as a stable personality trait. For example, recent studies in neuroscience suggest, among other brain areas such as the insula or the ACC, a role of the somatosensory cortices for empathy (e.g., when observing someone else being touched). Since the classic understanding of the primary somatosensory cortex is to represent touch on the body surface, we here aimed to test whether tactile performance is linked to the personality trait empathy. To test this, we examined the tactile acuity of 95 healthy participants (mean age 31 years) by using a two-point discrimination threshold task at the index fingers. Trait empathy was assessed by employing the interpersonal reactivity index (IRI), which measures self-reported empathy with four scales (empathic concern, perspective taking, fantasy, and personal distress). Results of regression analyses suggested the subscale empathic concern to be positively associated with performance in the tactile acuity task. We discuss this finding in the light of recent studies on empathy and consider possible implications of tactile training to enhance empathy.

## Introduction

Empathy describes our ability to understand others and interact with them. Although research still lacks a clear single definition of empathy, theoretical conceptualizations usually argue that empathy involves both cognitive as well as affective components, thereby enabling the individual to vicariously experience the feelings and understand the given situation of another (Hoffman, 2001; Neumann et al., 2015). For example, the perception-action model (PAM) suggests the ability to imagine a situation from the other person’s point of view and the sharing of emotions (de Waal and Preston, 2017). Moreover, the PAM suggests that empathy is based on a neural overlap between motor and affective representations of self and other (similar to the mirror neuron theory; Rizzolatti and Caruana, 2017). Interestingly, the PAM also includes “person-specific, conceptual, associative and affective representations to the overlapping representations of self and other” (Preston and de Waal, 2017). The present article is based in particular on this simulation-based model of empathy. Given that people who feel empathic or compassionate in a given situation help more often, it seems to be important to develop training opportunities that enhances our capacity to feel for another person (Klimecki et al., 2014; Singer and Klimecki, 2014).

The recent years have shown a growing interest in the understanding of the neural underpinnings of empathy (Singer et al., 2004; Lamm et al., 2011; Banissy et al., 2012). Most researchers agree on the main network including ACC and anterior insula for state empathy, e.g., when witnessing somebody in pain (Singer et al., 2004). In addition to these brain areas, the involvement of primary (SI) and secondary (SII) somatosensory cortices has also been reported and linked to state (Bufalari et al., 2007) and trait empathy (Avenanti et al., 2009). Moreover, research demonstrated activation of somatosensory cortices when observing non-painful touch, too (Keysers et al., 2004; Blakemore et al., 2005; Schaefer et al., 2009, 2012; Kuehn et al., 2013). Recent studies report that the magnitude of this vicarious activation predicts interindividual differences in empathy (Gazzola et al., 2006; Schaefer et al., 2012). A role for the somatosensory cortices is also suggested by recent findings in autism spectrum disorders (Khan et al., 2015).

Previous studies have also tried to address the different neural substrates for affective and cognitive components of trait empathy (e.g., Shamay-Tsoory et al., 2009). Using voxel-based morphology, Banissy et al. (2012) examined gray matter volume and found differences in the precuneus, anterior cingulate, somatosensory cortex, and insula for affective empathy (empathic concern and personal distress), while the anterior cingulate and dorsolateral prefrontal cortex seem to be important for the cognitive component of empathy (perspective taking and fantasy; Banissy et al., 2012). Furthermore, a recent study suggested that the different parts of empathy seem to be associated with markers of myeloarchitectural integrity in the insular and somatosensory cortex (Allen et al., 2017). Additionally, it has been demonstrated that affective empathy (personal distress) is linked to both behavioral and electrophysiological responses (alpha/mu-rhythm) to observed social touch (Peled-Avron et al., 2016). Peled-Avron et al. (2016) examined participant’s responses when viewing pictures showing social touch. They found that individuals rated touch depicting photos as inducing more pleasant emotions than pictures not showing touch. This effect was more pronounced in participants with high scores on the PD subscale (but not with the other dimensions of the IRI). Furthermore, PD (as well as PT) predicted the amount of mu suppression for observed touch.

The above-mentioned studies suggest a role of the somatosensory cortices for state and trait empathy. Interestingly, the classical understanding of SI is to represent touch applied on the body surface in a more or less mechanical way (Kaas, 2008). For example, it is well known that tactile performance can be linked to an engagement of the somatosensory cortices (e.g., Schmidt-Wilcke et al., 2018). The studies reported above challenge this view and argue that the somatosensory cortices may also be important for processes related to empathy.

In the present study, we aimed to examine the role of somatosensation in empathy by testing whether interindividual differences in empathy are linked to tactile performance. Since both tactile acuity (or sensitivity) performance, as well as trait empathy seem to engage the somatosensory cortices (vicarious activation of SI is stronger for more empathic people; Schaefer et al., 2012; Peled-Avron et al., 2016), we hypothesized that empathic personality traits may be associated with the performance in a tactile acuity task. Thus, we assumed that more empathic participants show better results in a tactile performance task.

How could empathy be related to tactile performance? We hypothesize that empathic individuals may express stronger attention not only to other’s human sensations but also to their own sensations. Therefore, higher empathy levels might facilitate tactile acuity by top-down processes. A possible way would be that empathic personality traits might affect somatosensory cortices *via* the insula. The insula is described as a neural substrate for state empathy, but also known as an interface for attention-related processes (Singer et al., 2004; Lamm et al., 2011) as well as for the awareness of tactile information (Burton and Sinclair, 2000; Duncan and Boynton, 2007; Craig, 2009). Thus, attention (driven by empathy) might affect somatosensory function *via* the insula.

To test the link between empathy and tactile perception, we employed a tactile task using the 2-point-tactile discrimination threshold (2pd) and measured empathy personality traits by applying a self-report questionnaire in healthy participants.

Previous studies also report an interaction between empathy and age. However, the exact relationship remains to be cleared. While some studies found that trait empathy rises with age, other studies found the opposite result (Wieck and Kunzmann, 2015; Riva et al., 2018; Sun et al., 2018). Furthermore, tactile acuity or sensitivity seems to be reduced by age (Decorps et al., 2014; Wieck and Kunzmann, 2015; Zingaretti et al., 2019). Also, numerous studies discuss gender effects in trait and state empathy, suggesting higher empathy scores for females (Christov-Moore et al., 2014). Moreover, it has been reported that tactile acuity may be better in females (Peters et al., 2009). Therefore, we included sex and age as variables in our analyses.

Given that it is well-known that tactile training can improve tactile acuity, we also controlled the variable education, which may point to different lifestyles that might have influenced tactile performance in our task (Ragert et al., 2004; Kerr et al., 2008; Mueller et al., 2019).

## Materials and Methods

### Participants

Ninety-five right-handed subjects (54 females, mean age 31.61 SD ± 7.91 years) with no previous history of psychological or neurological disorders or any known hand or head injuries participated in the study. Participants gave informed written consent to the study, which adhered to the Declaration of Helsinki and was approved by the local human subjects’ committee.

Nine of the subjects stated to have a high school degree, 69 had a university degree, and 14 participants claimed to be trained for specific jobs. Forty-eight participants worked in the services sector, 15 subjects in the social domain, and five as an employee in an office (residual participants did not answer this question).

The datasets generated during the current study are available from the corresponding author on reasonable request.

### Procedure and Instruments

We recruited participants at local universities, who were offered participation in this study by an online scheduling system. Besides, we also included non-university participants, which were found by flyers and social media in the local area. Participants were not included when neurologic disorders were known or when age was below 18 or above 60 years. All participants were then asked to first perform the tactile performance task and then to complete the IRI questionnaire.

We used a custom-made device to assess 2pd thresholds. This device was based on a commercially available discriminator, which was only slightly changed by adding additional needles to provide more possibilities to test subjects (AFH-Webshop, Lügde, Germany)^1^. The device consisted of seven pairs of brass needles mounted on a rotatable disc that allowed switching rapidly between pairs. Spacing between pins ranged from 1 to 4 mm. A single needle was used as the control condition. The needles were applied at the tip of the left and right index fingers (D2) as previously described (Pleger et al., 2001; Philipp et al., 2015). Participants had to close their eyes prior beginning of the testing. The stimuli were presented ten times in randomized order. The participants were not informed about the ratio of needle pairs and single needles. Each session consisted of 80 trials. After each trial participants had to decide immediately after application if they had felt one or two sensations. We then calculated the number of correct responses for all trials to receive a score of tactile performance acuity.

Trait empathy was examined by using a German version of the IRI (Davis, 1983; Paulus, 2009). The IRI is an established questionnaire of self-reported empathic behavior. It is widely used in different contexts and extensively validated (e.g., Singer et al., 2004; Avenanti et al., 2009; although it has also been criticized, e.g., Jolliffe and Farrington, 2006). The 28-item survey consists of four subscales with each pointing to a different aspect of empathy. The scale perspective taking (PT) refers to the tendency to cognitively imagine a situation from the other person’s point of view. The scale FS (fantasy) reflects the tendency of individuals to transpose themselves into the feelings and actions of fictional characters in books, movies, or plays. Empathic concern (EC) assesses feelings of sympathy and concern for others. The scale personal distress (PD) measures the tendency to feel distressed or unease in response to distress in others. Davis describes EC and PD as the affective component, whereas PT and F should measure the cognitive component of empathy (Davis, 1983).

### Statistical Analyses

To test our hypothesis, empathy personality traits of the IRI went into standard multiple linear regression analyses to analyze the relationship with the tactile performance of the right index finger. As additional predictors, we employed age and sex (as a dummy variable). All four empathy dimensions (as well as age and gender) went simultaneously into one regression model. Furthermore, we performed stepwise regression models, in which the same predictors went not simultaneously but in a stepwise order into the model. Analog regression analyses were performed for tactile acuity of the left index finger, again with all four empathy dimensions (and sex and age) as simultaneous predictors. Last, we computed a regression model for both left and right tactile acuity.

The software package SPSS was used for all statistical analyses (IBM Corporation, Armonk, NY, USA).

## Results

Table 1 depicts the mean scores for IRI subscales. EC correlated significantly with FS (*r* = 0.52, *p* < 0.01) and PT (*r* = 0.41, *p* < 0.001). PT was linked to FS (*r* = 0.31, *p* < 0.01; corrected for multiple comparisons; Table 2).

**Table 1:** Results of empathy personality questionnaire interpersonal reactivity index (IRI).

Empathy personality questionnaire IRI	Females (mean ± standard deviation)	Males (mean ± standard deviation)
Empathic concern	16.21 ± 2.20	15.05 ± 2.62
Personal distress	10.74 ± 2.70	10.26 ± 2.32
Perspective taking	15.94 ± 2.48	14.81 ± 3.11
Fantasy	14.85 ± 3.04	13.38 ± 2.84

**Table 2:** Correlation matrix of tactile performance with empathy personality questionnaire IRI.

	EC	PD	PT	F	Tactile acuity right hand	Tactile acuity left hand
Empathic concern (EC)						
Personal distress (PD)	0.02					
Perspective taking (PT)	0.41*	0.01				
Fantasy (FS)	0.52*	0.15	0.31*			
Tactile acuity right hand	0.25*	−0.03	0.15	0.05		
Tactile acuity left hand	0.20	0.03	0.16	0.13	0.69*	

Pearson, *correlation is significant at 0.01 level.

Females showed higher empathy scores for most of the IRI subscales with small or medium effect sizes (EC: *t*_(93)_ = 2.35, *p* = 0.02, Cohen’s *d* = 0.48; FS: *t*_(93)_ = 2.41, *p* = 0.02, Cohen’s *d* = 0.50; PT: *t*_(93)_ = 1.98, *p* = 0.05, Cohen’s *d* = 0.41; PD: *t*_(93)_ = 0.91, *p* = 0.37). There were no other significant correlations.

Mean performance in the tactile task was 77.66% (standard deviation ±10.58) correct responses for right and 79.42% (±8.83) for the left index finger. More in detail, the mean performance was 23% for the smallest interval (1 mm), 42% for the interval of 1.5 mm, 78% for 2 mm, 94% for 2.5 mm, and almost perfect for bigger intervals and single needle (99%).

To examine relationships of empathy with tactile acuity of the right index finger we calculated a linear regression analysis, in which all four empathy dimensions (EC, PD, PT, FS), age, and sex were entered simultaneously into the model. The model showed a trend for significance (*R* = 0.35, adj.*R*^2^ = 0.06, *F*_(6,94)_ = 2.03, *p* = 0.07). The empathy score EC was a significant predictor for tactile acuity of the right finger (*β* = 0.33, *p* = 0.01), whereas FS, PD, and PT failed to show significant effects. Furthermore, age was a predictor at border of significance (*β* = 0.21, *p* = 0.05). Sex had no influence (*β* = 0.04, *p* > 0.10).

**Figure 1:**
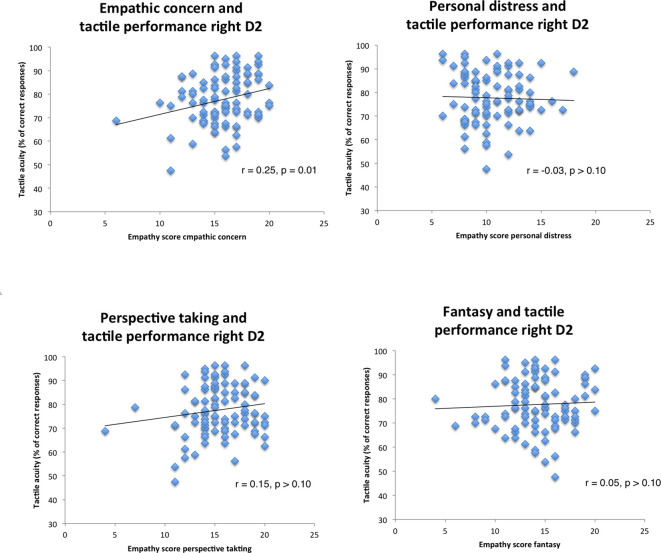
Correlation scatterplots for empathy scores of Interpersonal reactivity index (IRI) with tactile performance (right index finger, Pearson correlations).

Since our participants varied concerning the educational degree, we also tested education as a further predictor in our model (EC, PD, PT, FS, age, sex, and education were entered simultaneously into the model). Linear regression analysis showed a slightly improved model (*R* = 0.38, adj.*R*^2^ = 0.08, *F*_(7,94)_ = 2.11, *p* = 0.05) and confirmed EC as a significant predictor (*β* = 0.33, *p* = 0.01; see Table 3 and Figure 1). Educational degree was not a significant predictor (*β* = 0.17, *p* > 0.10). There were no other significant predictors.

**Table 3:** Regression analyses of left and right 2-pD threshold with empathy subscales as predictors.

2-pDT	Model					Coefficients (standardized)		
	*R*	*R*^2^	adj. *R*^2^	ANOVA		*β*	*T*	Sign
					EC:	0.33	2.70	***p* = 0.01**
					PD:	0.05	0.44	*p* = 0.83
					PT:	0.07	0.63	*p* = 0.66
Right D2	0.38	0.15	0.08	*F*_(7,94)_ = 2.11, *p* = 0.05	FS:	−0.10	−0.83	*p* = 0.41
					Age:	0.17	1.56	*p* = 0.12
					Educ.:	0.17	1.55	*p* = 0.13
					Sex:	0.02	0.21	*p* = 0.84
					EC:	0.17	1.32	*p* = 0.19
					PD:	0.04	0.33	*p* = 0.74
					PT:	0.08	0.65	*p* = 0.52
Left D2	0.28	0.08	0.00	*F*_(7,94)_ = 1.04, *p* = 0.41	FS:	−0.01	−0.08	*p* = 0.94
					Age:	0.15	1.33	*p* = 0.19
					Educ.:	−0.06	−0.51	*p* = 0.61
					Sex	−0.10	−0.91	*p* = 0.37

*All four IRI dimensions (EC, F, PT, PD, age, sex, and education) went simultaneously in one model. Significant values in bold*.

To further analyze the variance of the predictors we employed a stepwise regression model using the same predictors. The stepwise regression started with zero predictors and then added the strongest predictor to the model, then the second strongest predictor, and so on. If this procedure resulted in changing the significance of a previously entered predictor, the procedure removed it from the model (stepwise selection using *F*-probabilities, the threshold for inclusion was 0.05, for exclusion 0.10). Results suggested two significant models with either EC (*β* = 0.25, *p* = 0.01) or EC and educational degree as significant predictors (EC, *β* = 0.29, *p* = 0.005; educational degree, *β* = 0.22, *p* = 0.03). All other variables (FS, PD, PT, age, gender) were excluded. For all these linear regression analyses multicollinearity was low (all VIF scores below 1.03).

Visual inspection of the scatterplots identified two outliers (see Figure 1, empathic concern and right D2, left and bottom). When removing these two outliers the results still hold (EC: *β* = 0.25, *p* = 0.04). To further test whether some outliers drive the correlation, we performed a robustness check by employing a regression model using bootstrapping (1,000 samples, 95% confidence intervals, percentile method). Again, all four empathy dimensions (EC, PD, PT, FS), age, educational degree, and sex went simultaneously into the model. Results confirmed our findings by demonstrating that the empathy score EC was a significant predictor for tactile acuity of the right finger (EC: *p* < 0.01). Other empathy dimensions (FS, PD, PT) or variables failed to show significant effects (see Table 4).

**Table 4:** Robust regression analyses (bootstrapping) of left and right 2-pD threshold with empathy subscales as predictors.

2-pDT			Bootstrap	
		Sign.	95% confidence interval (lower/upper endpoints)	
	EC:	***p* < 0.01**	0.20	1.20
	PD:	*p* = 0.64	−0.49	0.78
	PT:	*p* = 0.57	−0.52	0.99
Right D2	FS:	*p* = 0.36	−0.88	0.32
	Age:	*p* = 0.08	−0.09	0.42
	Educ.:	*p* = 0.11	−0.87	4.86
	Sex:	*p* = 0.87	−3.27	3.99
	EC:	*p* = 0.18	−0.26	1.22
	PD:	*p* = 0.74	−0.46	0.67
	PT:	*p* = 0.58	−0.48	0.81
Left D2	FS:	*p* = 0.93	−0.56	0.49
	Age:	*p* = 0.15	−0.02	0.35
	Educ.:	*p* = 0.56	−3.11	1.60
	Sex	*p* = 0.39	−4.66	1.87

*All four IRI dimensions (EC, F, PT, PD, age, educational degree, and sex) went simultaneously in one model. Significant values in bold*.

**Figure 2:**
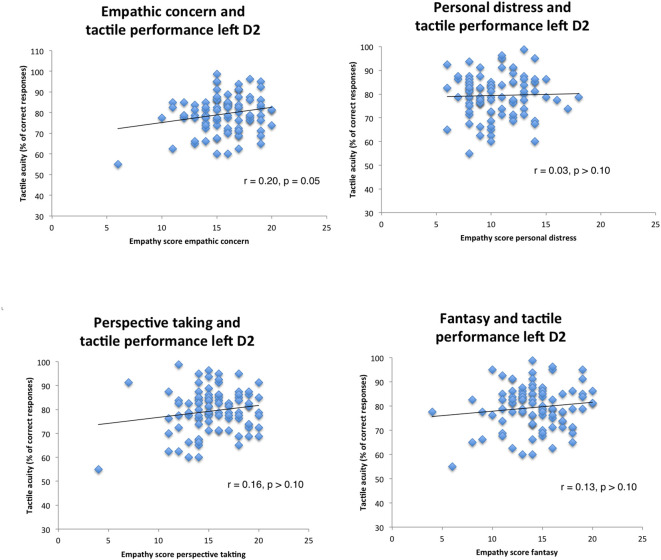
Correlation scatterplots for empathy scores of IRI with tactile performance (left index finger, Pearson correlations).

We then examined possible relationships of empathy personality traits with the left index finger. We computed a regression model in which all predictors went simultaneously into the analysis (EC, FS, PD, PT, gender, education, and age), analogous to the previous calculation. Results revealed no significant predictors (all *p*’s > 0.10, see Figure 2; *R* = 0.27, adj.*R*^2^ = 0.01, *F*_(6,94)_ = 1.18, *p* > 0.10).

We finally calculated a regression model for tactile acuity in general (mean of left and right D2 scores). The model failed to reach the level of significance (*R* = 0.33, adj.*R*^2^ = 0.04, *F*_(7,94)_ = 1.53, *p* = 0.17).

## Discussion

Based on recent findings that suggested the role of the somatosensory cortices (SI and SII) for empathy, the present study aimed to test the hypothesis that tactile perception ability is linked with trait empathy. Our results showed that tactile perception acuity is associated with interindividual differences in the empathy subscore EC.

Previous research has already suggested that performance in tactile tasks might be related to personality traits. These studies focused in particular on the theory of Eysenck. In his theory Eysenck suggested a link between cortical arousal and sensitivity, hypothesizing that “arousal messages” from the ARAS and the visceral brain may facilitate the detection of weak stimuli by raising the cortical arousal (Eysenck, 1967). Psychophysiological studies found some support for this theory. For example, Edman et al. reported lower tactile detection thresholds in introverts (Edman et al., 1979). Also, it has been shown that somatosensory evoked potentials (SEPs) were associated with extraversion (Shagass and Schwartz, 1965). The present results also report a link between personality and tactile sensitivity, but our results point to a role for empathy (EC) as a personality trait that may be linked with our tactile performance.

Interestingly, we only found a link between tactile performance and EC, not for the other empathy subdimensions. According to Davis (1983), empathy can be divided into an affective and a cognitive part. Affective empathy is described by ED and PD. PD reflects the tendency to feel distressed in response to distress in others. It’s role in empathy is controversially discussed (Davis, 1983). EC is characterized by feelings of sympathy and concern for others. Our results support the view that empathy can be differentiated into at least two parts, whereas only the emotional dimension seems to be linked to tactile performance.

Our results are supported by a similar experiment reported by Philipp et al. (2015). The authors describe an experiment in which a Zen meditation exercise resulted in an enhanced tactile acuity. Experienced Zen scholars were asked to meditate for 3 days for at least 8 h. During these meditation exercises, the participants practiced focused-attention meditation, which is characterized by focusing sustained attention on an object. Here the task was to “be completely aware of the spontaneously arising sensory perception in their right index finger” for periods of 2 h. A control group of scholars practiced open-monitoring meditation, in which meditation was nonspecific and without focusing attention on an object. After 3 days the authors found enhanced tactile discrimination thresholds (measured with 2pd) for the focused attention group. The authors conclude that mental states can alter our tactile perception. We argue that the present study describes similar results. While Philipp et al. (2015) describe a link between meditation and tactile acuity, we here report a relationship between trait empathy and tactile acuity. Several studies report training that aims to improve empathy (or compassion) based on meditation (e.g., Klimecki et al., 2014). Thus, meditation (paying attention to our own body) may be a way to improve empathy [paying attention to (the body of) others].

Our results can also be related to a study reporting enhanced tactile sensitivity in mirror-touch synesthesia (Banissy et al., 2009). Banissy et al. (2009) found that synesthetes who experience touch have enhanced tactile sensitivity. Similarly, synesthetes who experience color have enhanced color sensitivity, thereby suggesting a relationship between the modality of synesthetic experience and the modality of the sensory enhancement. Banissy et al. (2009) conclude that a core property of synesthesia seems to be a “hyper-sensitive concurrent perceptual system.” A hyper-sensitive tactile system for highly empathic individuals (at least concerning the EC-dimension) may also account for our findings.

But why should empathic feelings be linked to tactile performance abilities in our fingertips? Our results may be interpreted in light of recent views of empathy as a process of simulation. According to Rizzolatti et al. (2001), we “understand others through an internal act, that recaptures the sense of their acting.” In this internal act we understand others by simulating other’s actions, sensations, or pain (see also PAM model; de Waal and Preston, 2017). Thus, whenever we see someone in pain or simply be touched, we understand the touch by a vicarious activation of our own somatosensory cortices. The present result might extend these thoughts by suggesting that the more accurate we are to our own sensations (tactile performance acuity), the more we are likely to be attuned to other’s bodily sensations (possibly through a process of simulation; Gazzola et al., 2006; Schaefer et al., 2012). Hence, we suggest that the empathic trait EC can be linked to tactile acuity through simulation processes.

Since the present experiment did not examine tactile acuity and vicarious sensations when observing someone else being touched, the link to simulation processes remains highly speculative. Additional experiments are needed to support the hypothesis that empathic individuals maintain a stronger activation of SI to simulate observed experiences, which then results in better performance in tactile tasks. Based on the present data we can only conclude that the higher the EC empathy level of an individual, the more this individual seems to be attuned to his or her bodily sensations.

Which mechanisms may drive the link between touch perception and the empathy subscore EC? Empathic individuals may express stronger attention both to other’s human sensations as well as to their own sensations. In this way, higher empathy levels might facilitate tactile acuity by top-down processes. But how may attention alter the own tactile sensations? Previous work demonstrated that the human somatosensory system covers more than 10% of the cortical surfaces, including not only SI and SII but also the insular cortex and other brain areas (Avanzini et al., 2016). Furthermore, recent studies suggest a ventral pathway of somatosensory perception (similar to the visual or auditory modality), originating from SI, passing the parietal operculum (SII) and terminating in the insular cortex (Dijkerman and de Haan, 2007; Preusser et al., 2015). This ventral stream has been related to the recognition and perception of tactile stimuli. We speculate that the insular cortex, which has been described as an interface for cognitive and affective processing, may be an important brain region for the shown link between empathy and tactile acuity in our study (Burton and Sinclair, 2000; Karnath et al., 2001; Duncan and Boynton, 2007; Craig, 2009). Given it’s known role in empathy, the insula might work as an interface for attention-related processes (which are linked to the personality trait empathy) and activity in SI (which represents performance in tactile acuity tasks; Duncan and Boynton, 2007). However, future studies are required to further examine the neural underpinnings of the link between trait empathy and tactile performance we here report.

Based on our data, it remains unclear if the link between the EC dimension of empathy and tactile acuity represents bottom-up or top-down processes. While the previous thoughts seem to focus on a top-down view, bottom-up processes may also explain our results. For example, individuals who are more attuned to their own tactile sensations may also be more attuned to the sensations of others. Hence, in this way, causality is the other way around. This does not seem unlikely, considering recent work on the causal role of SI in prosocial behavior, a key component of empathy (Gallo et al., 2018).

Our results suggest a possible relationship between EC empathy and tactile acuity limited to the right hand (all of our participants were right-handed). How do we explain this laterality of our results? Although we usually attribute emotions to the right side of our brain, this hemisphere is not exclusively responsible for processing emotional information. While the right hemisphere seems to be important for emotions linked to avoidance (e.g., fear), the left side may be associated with emotions related to approaching and engaging, such as happiness when seeing a smile (at least in right-handed individuals; Brookshire and Casasanto, 2018). We speculate that this approaching and engaging dimension may have driven the link between empathy and tactile acuity in our results. However, future studies are needed to explore this laterality effect.

There are several limitations to our study. For example, the main regression analysis of our results revealed only marginally significant results. Thus, we have to be careful with conclusions. Future studies are needed to replicate the results. Moreover, our sample is based on a predominantly non-university sample and relatively old participants. This limits the comparison to other studies, which are often based on younger and student populations. Previous empathy studies reported profound differences in participants of different ages. For example, O’Brien reported an inverse u-shaped pattern for empathy across the life span (O’Brien et al., 2013). Also, empathy was measured using a self-reporting questionnaire, which might have measured a self-description of our participants rather than their empathic personality. This self-reported empathy can be subject to biases such as social desirability (Obst et al., 2016). Furthermore, possible alternative explanations for our results should also be taken into account. For example, empathic participants may have focused more strongly on the task. However, given that previous studies do not report that empathy is linked to conscientiousness (Mooradian et al., 2011), we think that it is unlikely that the effects we report simply reflect attention to the task.

What are the implications of this study? We speculate that (although we here examined empathy as a relatively stable personality trait) future research might test whether a possible empathy training might be developed based on sensorimotor exercises. For example, individuals showing deficits in empathic feelings (e.g., psychopaths) might benefit from tactile training by enhancing the attention to their own and other’s bodies.

Taken together, our findings suggest that the empathy dimension EC may be associated with the tactile modality in a more direct way than previously thought. Thus, the often neglected tactile sense does not only provide us with information about what is going on our body surface, but the way we feel with our own body might also be important for the perception of our social life.

## Data Availability Statement

The raw data supporting the conclusions of this article will be made available by the authors, without undue reservation.

## Ethics Statement

The studies involving human participants were reviewed and approved by Ethical Committee of Medical School Berlin. The patients/participants provided their written informed consent to participate in this study.

## Author Contributions

MS conceived and designed the experiment and wrote the article. MJ and NR performed the study. MJ, NR, and MS analyzed the data. All authors contributed to the article and approved the submitted version.

## Conflict of Interest

The authors declare that the research was conducted in the absence of any commercial or financial relationships that could be construed as a potential conflict of interest.

## Footnotes

^1^https://premium-therapie.de/de/befund-diagnostik/sensorik-senibilitaetstest/afh-2-punkt-diskriminator-duo
